# Basal-bolus regimen versus twice-daily premixed insulin in the treatment of childhood type 1 diabetes mellitus in Mosul City: A comparison study

**DOI:** 10.14341/probl13609

**Published:** 2026-03-07

**Authors:** I. A. Mohialdeen, A. H. AL-Numan

**Affiliations:** AL-Khansaa Maternity and Children Teaching HospitalИрак; AL-Khansaa Maternity and Children Teaching HospitalIraq; University of Mosul, College of MedicineИрак; University of Mosul, College of MedicineIraq

**Keywords:** Type 1 Diabetes Mellitus, basal-bolus insulin, Twice-daily insulin, Children, lipohypertrophy, Type 1 Diabetes Mellitus, basal-bolus insulin, Twice-daily insulin, Children, lipohypertrophy

## Abstract

**BACKGROUND:**

BACKGROUND: Type 1 diabetes mellitus is the most common endocrine-metabolic disorder in childhood and adolescence. Some families may find it difficult to administer four daily injections, especially in young children, or to use the newer, expensive insulin analogs and pumps. For this reason, many physicians are still using the classical two-injection schedule, using premixed insulin in certain areas of the world.

**AIM:**

AIM: To assess glycemic control and complication indicators in type 1 diabetic children on premixed or basal-bolus insulin.

**METHODS:**

METHODS: One hundred children aged 2–14 years with type 1 diabetes mellitus were studied at multiple diabetes care centers; fifty were receiving premixed insulin, and the other fifty were on a basal-bolus insulin regimen. Evaluations were made based on HbA1c levels, occurrences of hypoglycemia, ketoacidosis, and other complications.

**RESULTS:**

RESULTS: The study revealed significant improvements in HbA1c levels in the basal-bolus insulin group compared to premixed insulin patients three and six months after treatment (p=0.048 and p=0.005, respectively). Patients using the premixed regimen experienced more frequent hypoglycemia attacks (p=0.001) and injection site complications, such as hypertrophy (p=0.001).

**CONCLUSIONS:**

CONCLUSIONS: It has been revealed that a basal-bolus regimen (MDI) improves children’s and teenagers’ glycemic control with fewer complications.

## Introduction

Insulin-dependent diabetes mellitus (IDDM) during childhood makes up 5–10% of all cases of diabetes [1]. It is considered the most common chronic disease among children all over the world [2].

Insulin regimens for people with type 1 diabetes (T1DM) should aim to simulate the normal physiologic secretion of insulin in people without diabetes. Intensive insulin therapy, which can be achieved with either a basal-bolus insulin regimen using multiple daily injections (MDI) or a continuous subcutaneous insulin infusion (CSII), is now the standard of care required at the onset of type 1 diabetes (T1DM), for people of all ages [3].

Isophane insulin, commonly referred to as Neutral Protamine Hagedorn (NPH) insulin, was introduced to the world for the first time in 1946 when Nordisk added zinc to protamine insulin, it extends its duration of action and improves blood sugar control making the drug even more appealing as it can be taken just twice every day when mixed with regular insulin[4]. The pharmacokinetics of the premixed insulins promote patient convenience and 24-hour effectiveness. This explains why premixed insulins have higher adherence rates and better glycemic control if compared to multiple daily injection regimens [5]. Neutral protamine Hagedorn (NPH) and regular human insulin are combined in 30/70 or 50/50 ratios to create conventional premixed biphasic human insulin. The effects of these endure for around 10 to 16 hours, and they are given 30 minutes before meals. The newer analog premixed formulations (Lispro to intermediate lispro protamine 25/75, or 50/50) and (aspart to intermediate aspart protamine 30/70, and 50/50) can be taken up to 15 minutes before meals and have a faster onset of effect. This newer formulation has a longer duration of action, ranging from 12 to 24 hours, as compared to traditional premixed preparations [6].

In 1993, the Diabetes Control and Complications Trial demonstrated that intensive insulin therapy for T1DM decreases the risk of microvascular complications, outweighing the increased risk of hypoglycemia associated with it. This goal is typically pursued using a multi-dose insulin regimen that combines basal and short-acting insulin. Long-acting analog insulins (detemir, glargine, and degludec) provide peak less background insulin lasting up to 24 hours although sometimes they need to be given twice daily except for degludec, while the short-acting insulin-like lispro, Aspart, and Glulisine usually given during the day time with meals to suppress postprandial blood glucose [7].

A basal-bolus regimen offers flexibility regarding meal timing and daily routines, while also minimizing the necessity for snacking. This approach is generally more suitable for older children and adolescents, while younger toddlers who consume multiple meals during the day may not be suitable for such a regimen [8].

In Japanese children and adolescents with IDDM, the substitution of older twice and thrice-daily insulin regimens with the newer basal-bolus insulin regimen using insulin analogs over the years significantly reduces the occurrence of hypoglycemic episodes alongside providing better glycemic control reflected by lower HbA1c means [9].

In countries with low resources limiting the availability of the newer insulin analogs and the methods for self-monitoring of blood glucose alongside poor education, the use of premixed insulin given twice daily is still the primary treatment option despite the reports of poor glycemic control and higher HbA1c means [10]. This limited access to analog insulins is reflected in government policies across the region, where 56% of Middle East and North Africa Region (MENA) countries provide only human insulin, and just 39% offer broader coverage that includes both human and analog formulations [11].

The purpose of this study was to compare the safety and effectiveness of the basal-bolus insulin regimen using multiple daily injections (MDI) versus the premixed twice-daily insulin in managing children with Type 1 Diabetes Mellitus.

## Patients and methods

## Study design

This was a retrospective cohort study done in a multicenter Diabetic care setting (Al-Wafaa Specialized Center for Diabetes and Endocrinology, Alkhansa’a Teaching Hospital diabetic clinic) from July 10, 2024, to January 1, 2025.

## Inclusion criteria

Any patients with diabetes mellitus type 1 who attended the abovementioned clinics, aged 2–14 years, who have been receiving insulin for the last 3 months, were included.

## Exclusion criteria

Less than 3 months elapsed since the diagnosis of DM, any patient who has changed insulin therapy regimen in the past 3 months, or any patient with additional disorders that may alter HbA1c readings (celiac disease, Addison disease, thyroid disease, thalassemia, sickle-cell disease).

## Study Population and Insulin Regimen Details

One hundred patients were included in this study, fifty on a premixed insulin regimen comprising 70% NPH and 30% Aactarapid. The NPH (Neutral Protamine Hagedorn, or isophane insulin) is an insoluble intermediate-acting insulin, while Actarapid is a short-acting insulin (Novo Nordisk). 2/3 of the mixture dose is usually given in the morning and 1/3 in the evening, 30 minutes before the meal. For the remaining fifty patients on a basal-bolus insulin regimen, the basal element constitutes 50% of the total dose and includes long-acting glargine (Lantus solo star-100 unit/ml — Sanofi-Aventis) which is approved for children from the age of 2 years, with rapid or short (soluble) acting insulin as bolus doses like aspart (NovoRapid-100 unit/ml — Novo Nordisk) or Lispro (Humalog-100 unit/ml — lilly) [12].

Since the onset of action of short-acting insulins is 5–15 minutes, they could be given shortly before or even after the main meals, and this can be helpful in certain situations when young children have a difficult time with meals [13]. Also, patients were advised to take an extra insulin dose before snacks (broadly 1 unit for each 10 g carbohydrate). No patient uses an insulin pump or has Continuous Glucose Monitoring.

The initial insulin regimen is often chosen based on the preference and judgment of the on-call physician, rather than following a set protocol. This decision is also shaped by which types of insulin are available at the center, as well as how prepared the patient and their family are to start newer insulin analogues, which require close monitoring and may come with financial challenges.

## Method

For all patients, a uniform questionnaire was applied which includes the following information: age, sex, residence, duration of DM, height, weight, body mass index, parental education, whether the mother is working or not, economic status, school absence, the coexistence of another disease (celiac disease, Addison, thyroid disease, thalassemia, sickle cell disease), type of insulin regimen used, weight changes in the last three months, early morning hypoglycemia, HbA1c levels 3 and 6 months after starting the basal-bolus regimen, and at the time of diagnosis, in addition to the number of hypoglycemic & diabetic ketoacidoses (DKA) attacks in the last 3 months. Regarding hypoglycemia, we categorized the attacks of hypoglycemia into mild defined as plasma glucose level less than 70 mg/dL but more than 54 mg/dl with symptoms like tremor, sweating, dizziness, hunger, irritability, or tachycardia, and severe attack defined as blood glucose concentration less than 54 with severe symptoms like Confusion, seizures, loss of consciousness, or inability to eat or drink [14].

Regarding complications, we asked the family about any complications related to diabetes mellitus (nephropathy, neuropathy, or retinopathy), atrophy, or hypertrophy at the site of insulin injection.

## Statistical Analysis

Data introduction, coding, and tabulation were performed via Microsoft Excel 365. Descriptive and analytic statistics were performed using the Minitab version 22 software statistical program. The descriptive statistics include mean ± Standard Deviation (SD) for measurable variables and frequencies and percentages for categorical variables. A Paired T-test of the two means was used for comparison between quantitative parameters. The improvement rate was calculated from the equation:

"% Improvement rate = [(before – after) / Before] × 100".

Whereas the chi-square test was performed for comparison between categorical variables. P-values ≤0.05 were considered statistically significant.

## Ethical considerations

This study was explained to each patient’s parent, and verbal consent was obtained from each guardian. Confidentiality of data was ensured.

Ethical approval was obtained from the Scientific and Ethical Research Committee at Nineveh Health Directorate number 257 on 3-7-2024.

## Results

At the time of diagnosis, the majority of patients had HbA1c levels indicative of poor glycemic control (>9%). However, a significant improvement was observed after three months of treatment with the basal-bolus insulin regimen (p=0.048), which became even more pronounced at six months (p=0.005) compared to those receiving the twice-daily premixed insulin regimen. By six months, most patients in the basal-bolus group achieved HbA1c levels below 7%, whereas the majority of those in the premixed group remained within the 7–9% range, as detailed in Table 1.

**Table:** Table 1. A comparison of basal-bolus and premixed insulin regimens in HbA1c levels among the studied children. *The chi-square test was used.

Parameters	Basal bolus regimen [ n=50]	Premixed regimen [ n=50]	P-value*
n.	%	n.	%
HbA1c at diagnosis	Normal <5.7%	0	0	0	0	0.882
Good control <7%	0	0	0	0
Fair (7–9 %)	13	26.0	14	28.0
Poor control (>9%)	37	74.0	36	72.0
HbA1c after three months	Normal <5.7%	0	0	0	0	0.048
Good control <7%	11	22.0	5	10.0
Fair (7–9 %)	32	64.0	29	58.0
Poor control (>9%)	7	14.0	16	32.0
HbA1c after 6 months	Normal <5.7%	1	2.0	1	2.0	0.005
Good control <7%	35	70.0	18	36.0
Fair (7–9 %)	10	20.0	27	54.0
Poor control (>9%)	4	8.0	5	10.0

Furthermore, the percentage reduction in HbA1c after three months was significantly greater in the basal-bolus insulin group (17.0%) compared to the twice-daily premixed insulin group (10.1%), as shown in Table 2. This improvement continued over time, with HbA1c reductions reaching 28.5% in the basal-bolus group and 18.1% in the premixed group after six months of therapy, as illustrated in Table 3.

**Table:** Table 2. Rate of improvements in HbA1c after three months among the studied children. * % Improvement rate = [(before – after) / Before] × 100.** A paired T-test of two means was used

Insulin regimen	HbA1c at diagnosis Mean±SD	HbA1c after three months of therapy Mean±SD	% Improvement after three months *	P-value**
Basal bolus regimen	10.0±0.8	8.3±1.2	17.0 %	0.001
Premixed regimen	9.9±0.9	8.9±1.2	10.1 %	0.001

**Table:** Table 3. Rate of improvement in HbA1c after six months among studied children. * % Improvement rate = [(before – after) / Before] × 100.** A paired T-test of two means was used.

Insulin regimen	HbA1c at diagnosis Mean±SD	HbA1c at last visit Mean±SD	% Improvement after six months	P-value**
Basal bolus regimen	10.0±0.8	7.1±0.8	28.5 %	0.001
Premixed regimen	9.9±0.9	8.1±0.6	18.1 %	0.001

Regarding demographic characteristics, children receiving the premixed insulin regimen were significantly more likely to reside in rural areas (60% vs. 30%, p=0.003) and to have parents with lower educational attainment, including a notably higher proportion of illiterate parents. Socioeconomic disparities were also evident, with a greater percentage of children in the premixed group coming from low-income families (48% vs. 22%, p=0.015). Additionally, a significantly larger proportion of mothers in this group were housewives (72% vs. 56%, p=0.001), as presented in Table 4.

**Table:** Table 4. Socio-demographic and family characteristics of the studied children. *The chi-square test was used.

Age	2–5 years	14	28.0	15	30	0.963
6–10 years	23	46.0	23	26
11–14 years	13	26.0	12	24
Sex	Male	17	34.0	23	46	0.221
Female	33	66.0	27	54
Residence	Urban	35	70.0	20	40	0.003
Rural	15	30.0	30	60
Father’s level of education	Illiterate	1	2.0	12	24	0.001
Primary	6	12.0	20	40
Secondary	14	28.0	11	22
Higher education	5	10.0	1	2
Postgraduate	24	48.0	6	12
mother’s level of education	Illiterate	2	4.0	14	28
Primary	6	12.0	17	34
Secondary	13	26.0	11	22
Higher education	6	12.0	2	2
Postgraduate	23	46.0	6	12
Mother’s occupation	Employee	22	44.0	14	28	0.001
Housewife	28	56.0	36	72
Economic status	Low	11	22.0	24	48	0.015
Moderate	29	58.0	22	44
High	10	20.0	4	8
School attendance	Yes	35	70.0	31	62	0.398
No	15	30.0	19	38

Children with longer diabetes duration (6–10 years) in this study use the premixed insulin regimen more frequently, whereas those with shorter duration of diabetes (1–5 years) generally utilize the basal-bolus insulin regimen, as seen in Figure 1.

**Figure:**
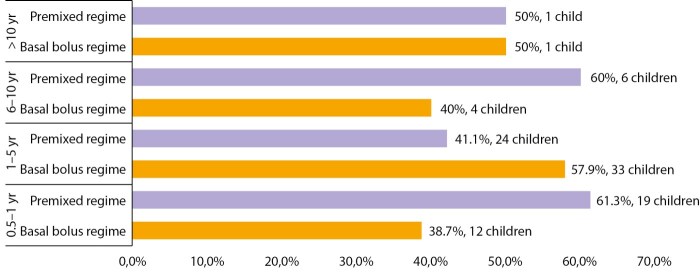
Figure 1. Distribution of duration of type 1 diabetes mellitus according to insulin regimen among the studied children.

As shown in Table 5, children in the premixed insulin group experienced a significantly higher frequency of hypoglycemic episodes — both mild and severe — compared to those in the basal-bolus group, except for early morning hypoglycemia. Moreover, injection site complications, including both atrophy and hypertrophy, were significantly more prevalent among patients receiving the premixed insulin regimen (p=0.001).

**Table:** Table 5. Type 1 DM complications in the studied children. *The chi-square test was used.

Variables	Basal bolus regimen [ n=50]	Premixed regimen [ n=50]	P-value*
n.	%	n.	%
Weight loss during the last 3 months	No weight loss	24	48.0	23	46.0	0.874
<3 Kg	19	38.0	21	42.0
3–5 Kg	7	14.0	6	12.0
Previous attack of hypoglycemia	None	39	78.0	16	32.0	0.001
Mild attack	5	10.0	21	42.0
Severe attack	6	12.0	13	26.0
Hospital admission for DKA	Absent	44	88.0	37	74.0	0.074
Present	6	12.0	13	26.0
Injection site status	Normal	41	82.0	20	40.0	0.001
Atrophy	1	2.0	3	6.0
Hypertrophy	8	16.0	27	54.0
Early morning hypoglycemia	Absent	44	88.0	47	94.0	0.295
Present	6	12.0	3	6.0

As shown in Table 6, none of the body measures (weight, height, and BMI) differed significantly between the two groups of children.

**Table:** Table 6. Comparison of weight, height, and body mass index among the studied children. *The chi-square test was used.

Weight (kg)	<10	1	2.0	1	2.0	0.099
10–19	12	24.0	19	38.0
20–29	11	22.0	15	30.0
30–39	19	38.0	7	14.0
≥40	7	14.0	8	16.0
Height (cm)	<100	5	10.0	3	6.0	0.485
100–119	10	20.0	16	32.0
120–139	19	38.0	21	42.0
140–159	12	24.0	8	16.0
≥160	4	8.0	2	4.0
Body mass index (kg/m2)	Underweight <5%	3	6.0	7	14.0	0.409
Healthy 5–85%	45	90.0	41	82.0
Overweight 85–95%	2	4.0	2	4.0
Obesity >95%	0	0	0	0

## Discussion

This study investigated the safety and effectiveness of the basal-bolus insulin regimen using multiple daily injections (MDI) versus the former standard premixed twice-daily insulin in managing children with Type 1 Diabetes Mellitus since many physicians still prefer to use the latter insulin regimen and are reluctant to change to newer protocols a practice still observed in countries such as china and new zealand [8][15].

All of the patients attending the diabetic clinic had high initial HbA1c that was in the range of fair to poor control; however, three and six months after the insulin treatment was initiated, there was a significant improvement observed in the basal-bolus group’s overall control of HbA1c. The rate of improvements in HbA1c indicated that the basal-bolus group significantly outperformed the premixed insulin regimen. The primary findings of this study were consistent with the conclusions of other studies conducted by Saleem (2023) in Baghdad [16], Sharef et al., (2015) in Oman [17], Shahid et al., (2016) in Pakistan [18], and Nathan et al. clinical trials [19][20].Conversely, Alabedi, (2020) [21] and Murphy & May Ng, (2013) [22] found that the basal-bolus regimen does not result in a significant lowering of HbA1c more than the conventional twice-daily insulin regimen, which may be explained by the poor adherence to the multiple daily injections required in this regimen and the small sample size.

In terms of hypoglycemic attacks, the premixed insulin regimen has a significantly higher attack rate than the basal-bolus regimen, which is explained by the need for strict timing of insulin injections and meals to avoid hypoglycemia, which is consistent with the systematic review by DeWitt & Hirsch, 2003, and researches conducted by Jabbari, Musleh et al. 2010 in Saudi Arabia, Chou et al., 2018 in China and Bellido et al. 2015 in Spain [23–26].While Alabedi (2020) in Baghdad concluded that no difference concerning hypoglycemia in either protocol exists, this contradiction might be explained by the lack of continuous monitoring of blood glucose daily [21].

Injection site complications especially hypertrophy were noticed more in the twice-daily insulin patients group in the current study, and this agrees with the results of a study done in India by Barola et al., 2018, and this can be explained by the larger amount of insulin delivered and the slower absorption of the older insulin preparations which will promote lipogenesis and hypertrophy [27].

There were no significant differences in the number of DKA episodes between the two groups, which contradicts a study by Chou et al., 2018 in China, in which the older regimen had more frequent episodes of DKA, and Alabedi, 2020 in Iraq, which concluded that DKA episodes were more among patients using the basal-bolus regimen, possibly due to the lack of adherence to multiple doses daily [21][25]. Also, no significant changes in weight or body mass index were observed in the last 3 months after using either regimen of insulin which aligns with the research conducted by Wang et al., 2023, in China. This lack of significant difference suggests that both insulin regimens have a similar impact on BMI, indicating that the choice between these regimens can be based on other factors such as patient preference, lifestyle, or specific Medical need rather than concerns about BMI changes alone [8].

In the current study, children on the premixed regimen are more likely to reside in rural areas, with a larger proportion of children coming from low-income families and a higher percentage of illiterate parents. This agrees with a study done by Alali & Afandi (2022) in Syria and Ewen et al. (2019) in the United States. This may be attributed to several factors, including limited access to healthcare resources, fewer diabetes care providers, and challenges in managing more complex insulin regimens like multiple daily insulin regimens, in addition to availability and being less expensive than the basal-bolus regimen [28][29].

These observations highlight how financial constraints, limited access to analog insulin, and insufficient infrastructure for frequent blood glucose monitoring may hinder the adoption of intensive insulin therapy in such settings. Also important to note is that implementing a basal-bolus regimen in our setting isn’t always straightforward. In real-life practice, the choice of insulin regimen often depends more on the individual physician’s judgment and what’s available at the center, rather than following a clear institutional protocol. The cost and availability of long-acting and rapid-acting insulin analogs, the need for more frequent blood glucose checks, and the family’s ability to afford and manage these demands all play a major role in shaping treatment decisions.

## Study limitations

## Confounding Variables

It’s challenging to control for all potential confounding factors that could influence the results, such as differences in patient adherence, lifestyle factors, or comorbid conditions.

## Data Completeness

The quality and completeness of historical data can vary, potentially leading to missing or inaccurate information.

## Causality

Retrospective studies can identify associations but cannot establish causality. Therefore, it’s difficult to determine whether the observed outcomes are directly due to the insulin regimen or other factors.

## Conclusions

The basal-bolus insulin regimen is superior to the premixed insulin regimen in controlling HbA1c in children and adolescents with type 1 diabetes mellitus but requires greater patient cooperation and frequent monitoring. It also reduces the number of hypoglycemic episodes and the local skin complications.
